# Typhoid during Anti-Typhoid Vaccination

**Published:** 1914-06

**Authors:** 


**Typhoid During Anti-Typhoid Vaccination.** J. Ferrand and R. Coville, Gaz. des Hop., page 1607, 1913. Their routine consisted in giving four injections of Vincent's multivalent vaccine at intervals of eight days. One person developed typhoid after the second injection, during the negative phase, but the attack was not serious. They do not regard the vaccination as a cause of the typhoid, 40 persons having been vaccinated without such an occurrence, in the same series and they naturally are sceptic as to the importance of the negative phase.

